# Vogt-Koyanagi-Harada Disease: A Case Report Through Poliosis and Inflammatory Relapses

**DOI:** 10.7759/cureus.111594

**Published:** 2026-06-27

**Authors:** Ziyu Wang, Celia Comberg, Dorine Makhoul, Abraham Beun, Aurelie Le

**Affiliations:** 1 Department of Ophthalmology, Centre Hospitalier Universitaire Saint-Pierre, Brussels, BEL; 2 Department of Internal Medicine, Centre Hospitalier Universitaire Saint-Pierre, Brussels, BEL

**Keywords:** corticosteroid, intravitreal injections, poliosis, posterior uveitis, uveitis, vogt-koyanagi-harada (vkh) disease

## Abstract

Vogt-Koyanagi-Harada (VKH) disease is a rare autoimmune stromal choroiditis affecting melanocyte-rich tissues and typically presenting with bilateral ocular inflammation associated with neurological, auditory, or integumentary manifestations. Early diagnosis is essential because prompt initiation of intensive immunosuppressive therapy is associated with improved long-term outcomes. An 18-year-old Hispanic man presented in September 2022 with a one-week history of bilateral visual loss and ocular redness. He had experienced a first episode of anterior uveitis in June 2022 and subsequently developed marked depigmentation of the hair, eyebrows, beard, and eyelashes beginning in July 2022. Ophthalmic examination and multimodal imaging demonstrated bilateral granulomatous panuveitis with active stromal choroiditis. Extensive infectious and inflammatory investigations were negative, while HLA-DRB1*04 testing was positive. A diagnosis of incomplete VKH disease was established. Despite treatment with intensive topical corticosteroids, systemic corticosteroids, mycophenolate mofetil, adalimumab, methotrexate, and repeated intravitreal dexamethasone implants, the patient developed recurrent inflammatory relapses and evolved toward a chronic-recurrent disease course. This case highlights the diagnostic value of early poliosis as a clinical clue to VKH disease, even in the absence of neurological prodromal symptoms. It also illustrates the potential for rapid progression toward chronic-recurrent inflammation despite aggressive multimodal therapy, emphasizing the importance of early recognition, close monitoring, and sustained treatment adherence.

## Introduction

Vogt-Koyanagi-Harada (VKH) disease is a multisystem autoimmune disorder characterized by T-cell-mediated inflammation directed against melanocyte-rich tissues and presenting as a primary stromal choroiditis. The disease is strongly associated with *HLA-DR4* and HLA-DRB1*04 alleles. It predominantly affects individuals with greater skin pigmentation, including Asian, Hispanic, Native American, and Southeast Asian populations, and is less frequently encountered in Caucasian patients. The median age at onset is approximately 35 years, with a slight female predominance [1-4].

The revised diagnostic criteria established in 2001 classify VKH disease as complete or incomplete according to the presence of bilateral ocular involvement associated with neurological, auditory, and/or integumentary manifestations, after exclusion of other causes of uveitis and in the absence of prior ocular trauma or surgery [5].

The disease classically evolves through a prodromal stage characterized by neurological and auditory manifestations, followed by acute bilateral ocular inflammation and, in some patients, late integumentary findings such as vitiligo, alopecia, or poliosis [1,6]. Modern multimodal imaging techniques, particularly indocyanine green angiography (ICGA) and enhanced depth imaging optical coherence tomography (EDI-OCT), have improved the detection and monitoring of stromal choroidal inflammation and play a central role in contemporary VKH management [6,7].

We report the case of an 18-year-old patient with incomplete VKH disease in whom rapidly progressive poliosis represented a prominent early clinical clue leading to the diagnosis. This case is noteworthy because the patient lacked neurological or auditory prodromal manifestations, progressed rapidly toward a chronic-recurrent inflammatory course, and remained difficult to control despite intensive multimodal immunosuppressive therapy. The case highlights the importance of recognizing early integumentary manifestations and performing timely multimodal choroidal assessment to facilitate prompt diagnosis and treatment of VKH disease.

## Case presentation

**Figure 1:**
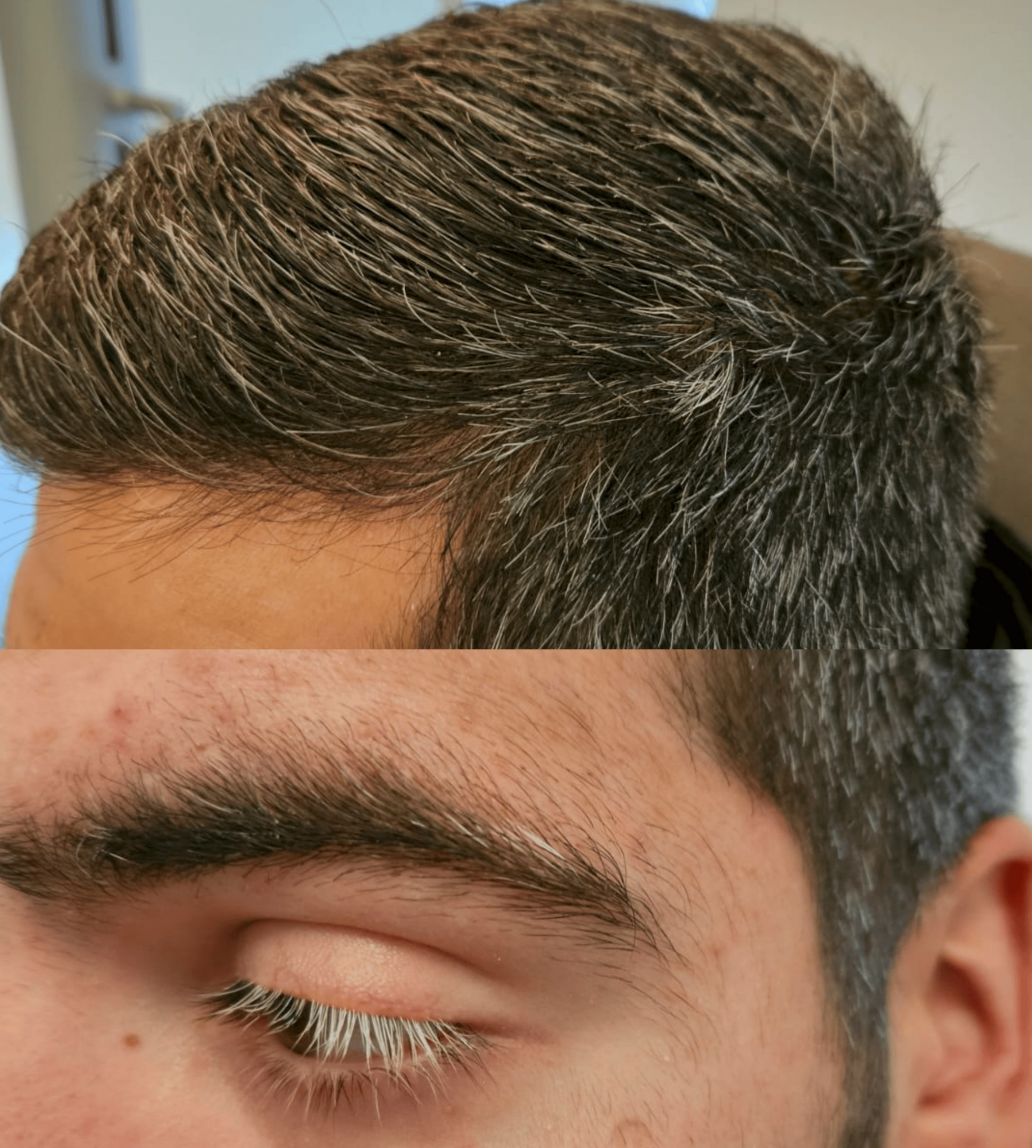
Poliosis of the patient’s eyelashes and hair.

An 18-year-old Hispanic man consulted our ophthalmology department at Saint-Pierre University Hospital in Brussels after a trip to Portugal for decreased vision and redness in both eyes; additionally, he had recently noted significant depigmentation of his hair, eyebrows, beard, and eyelashes (Figure 1). He had no neurological or auditory symptoms, and his ophthalmological history was unremarkable, except for a first episode of anterior uveitis several weeks prior. This first episode of anterior uveitis had been treated with topical steroids during his stay in Portugal and was then followed by the development of poliosis.

**Figure 2:**
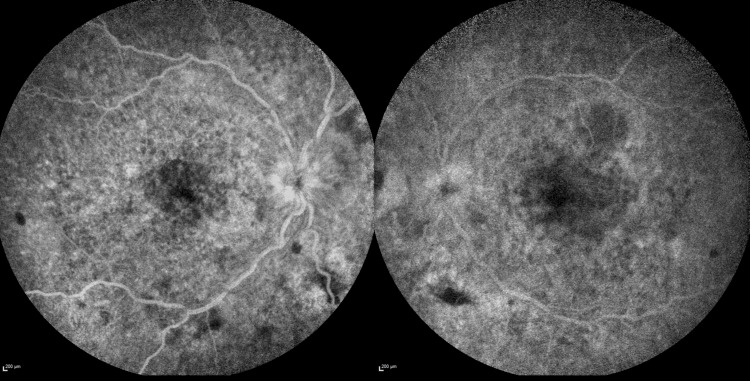
Early‑phase fluorescein angiography of both eyes demonstrating multifocal and diffuse pinpoint hyperfluorescent leaks, early pooling in areas of serous retinal detachment, and diffuse optic disc hyperfluorescence indicating active papillitis. Scattered hypofluorescent dark areas are also visible, consistent with choroidal granulomas or focal choroidal hypopefusion. These findings reflect active stromal choroiditis characteristic of acute Vogt-Koyanagi-Harada disease.

At presentation, best-corrected visual acuity (BCVA) was 16/20 in the right eye and 12/20 in the left eye. Intraocular pressure was normal in both eyes. Slit-lamp examination demonstrated bilateral granulomatous panuveitis with anterior chamber inflammation, posterior synechiae, vitritis, and Dalen-Fuchs nodules. OCT showed intraretinal cystoid changes associated with increased subfoveal choroidal thickness. Fluorescein angiography revealed multifocal pinpoint leakage, pooling in areas of serous retinal detachment, and optic disc hyperfluorescence (Figure 2). ICGA demonstrated numerous hypocyanescent dark dots consistent with active stromal choroiditis.

Based on the clinical presentation, differential diagnoses included sarcoidosis, tuberculosis, syphilis, sympathetic ophthalmia, and birdshot-like chorioretinopathy. Diagnostic investigations included chest computed tomography (CT), brain magnetic resonance imaging (MRI), interferon-gamma release assay, syphilis serology, Lyme serology, *Bartonella* serology, cytomegalovirus serology, herpes simplex virus serology, varicella-zoster virus serology, antinuclear antibodies, antineutrophil cytoplasmic antibodies, angiotensin-converting enzyme levels, HLA-B27 testing, and *HLA-DRB1* typing.

No infectious or alternative inflammatory cause was identified. Chest CT and brain MRI were normal. HLA-DRB1*04 testing was positive. Based on the presence of bilateral ocular involvement and integumentary manifestations in the absence of neurological symptoms, a diagnosis of incomplete VKH disease was established according to the revised diagnostic criteria.

**Figure 3:**
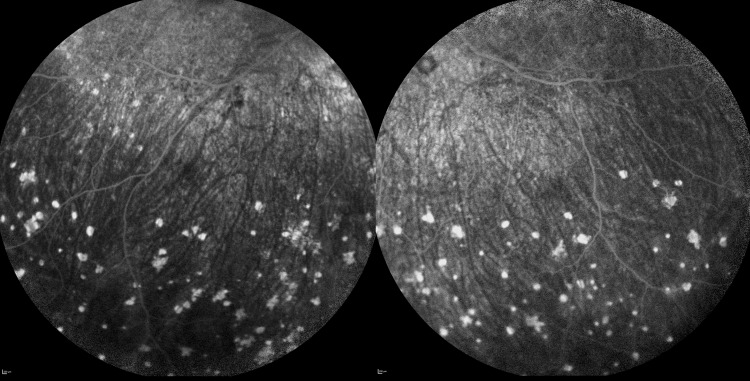
Late‑phase fluorescein angiography at the five‑month follow‑up showing numerous peripheral hyperfluorescent spots consistent with retinal pigment epithelium alterations and sequelae of prior inflammatory activity. No active pinpoint leakage is observed, indicating absence of acute Vogt-Koyanagi-Harada‑related inflammation.

**Figure 4:**
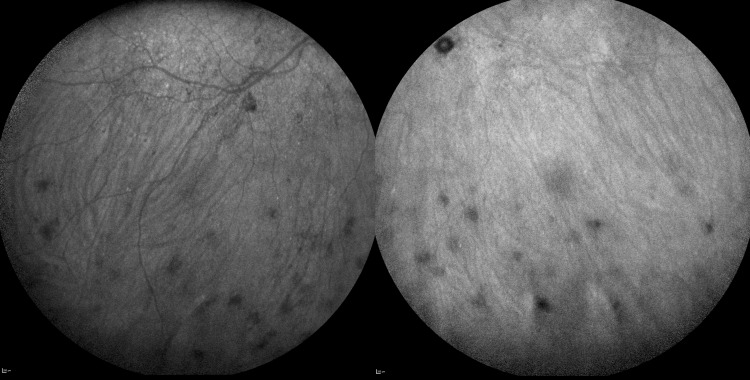
Mid-to-late phase indocyanine green angiography at the five-month follow-up demonstrating multiple persistent hypocyanescent spots in the periphery, corresponding to active choroidal granulomas. These findings reflect ongoing low-grade stromal choroidal inflammation.

Treatment was initiated with hourly prednisolone acetate 1% eye drops (Pred Forte®), dexamethasone 0.1% ointment (Maxidex®) at night, and tropicamide 0.5% three times daily. Although partial improvement was observed, persistent ocular inflammation prompted the initiation of oral methylprednisolone 48 mg/day in October 2022 (Figures 3, 4). Tapering was planned at a rate of 8 mg/week toward a maintenance dose of 8 mg/day. However, inflammatory recurrence occurred during tapering at 32 mg/day, leading to the introduction of mycophenolate mofetil at the end of October 2022.

Persistent inflammation despite combined corticosteroid and mycophenolate therapy resulted in the introduction of adalimumab in February 2023 (80 mg loading dose followed by 40 mg every two weeks). Because inflammation remained active, bilateral intravitreal dexamethasone implants (Ozurdex®) were administered in March 2023.

**Figure 5:**
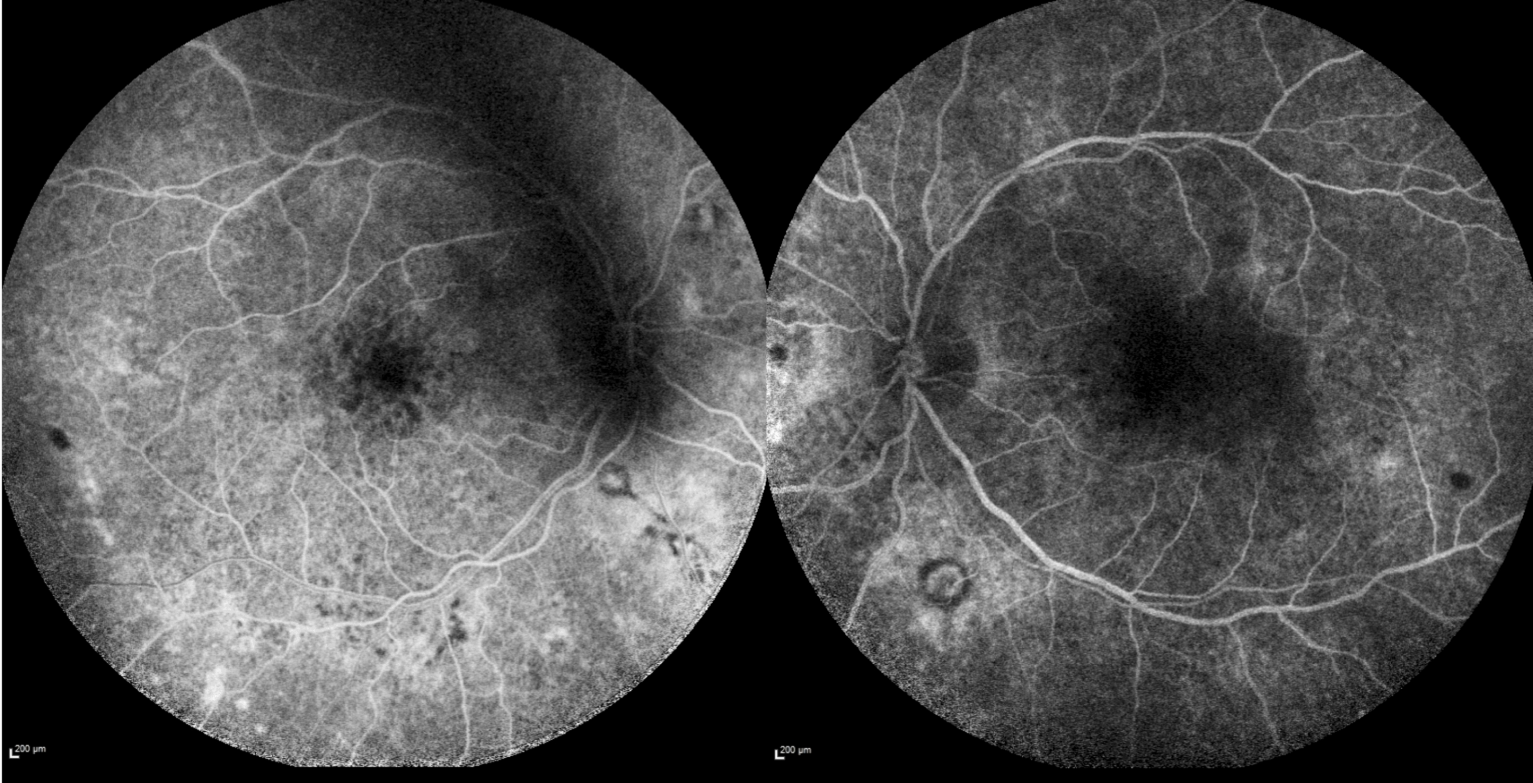
Early‑phase fluorescein angiography at the 15‑month follow‑up showing mottled hyperfluorescent and hypofluorescent retinal pigment epithelium alterations at the posterior pole, consistent with chronic Vogt-Koyanagi-Harada sequelae. No active pinpoint leakage or pooling is observed, indicating absence of acute inflammatory activity. Retinal vasculature appears intact, and optic disc staining is minimal.

**Figure 6:**
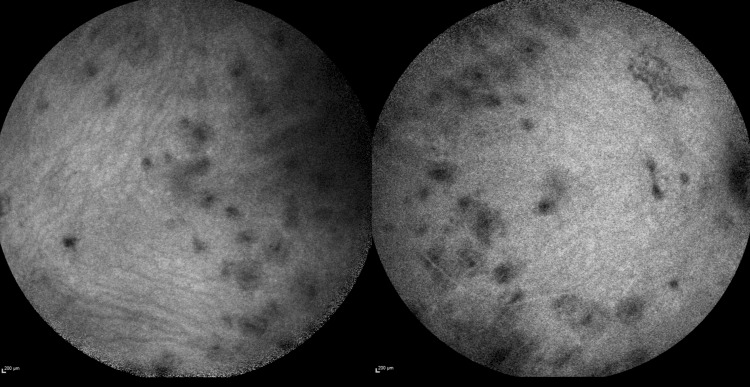
Mid-to-late phase indocyanine green angiography at the 15-month follow-up demonstrating numerous persistent hypocyanescent spots of varying size across the posterior pole and mid-periphery, corresponding to residual choroidal granulomas. Their persistence at 15 months suggests ongoing low-grade stromal choroidal involvement characteristic of chronic-recurrent Vogt-Koyanagi-Harada disease, in contrast to inactivity on fluorescein angiography.

Temporary inflammatory control was achieved, with BCVA improving to 20/20 in both eyes. Nevertheless, recurrent inflammatory flare-ups occurred during follow-up, characterized by recurrent anterior chamber inflammation, vitritis, and increased numbers of hypocyanescent lesions on ICGA (Figures 5, 6). Several relapses were temporally associated with poor adherence to adalimumab therapy.

Because of persistent disease activity, mycophenolate mofetil was replaced by oral methotrexate in December 2023, initially at 10 mg/week and subsequently increased to 15 mg/week. Repeated dexamethasone implants were required to control inflammatory exacerbations but were complicated by bilateral ocular hypertension.

At the last follow-up visit in February 2024, BCVA was 20/20 in the right eye and 18/20 in the left eye. Despite ongoing therapy with adalimumab, methotrexate, and low-dose corticosteroids, evidence of chronic-recurrent inflammatory activity persisted clinically and on ICGA. Table 1 presents a summary of the patient’s clinical course.

**Table 1:** Chronological summary of the clinical course.

Date	Clinical event
June 2022	First episode of anterior uveitis treated with topical corticosteroids in Portugal
July 2022	Onset of progressive poliosis involving scalp hair, eyebrows, beard, and eyelashes
September 2022	First presentation at our institution with bilateral granulomatous panuveitis; diagnosis of incomplete Vogt-Koyanagi-Harada disease established
October 2022	Oral methylprednisolone 48 mg/day initiated
October 2022	Mycophenolate mofetil introduced following inflammatory recurrence during corticosteroid taper
February 2023	Adalimumab initiated
March 2023	Bilateral intravitreal dexamethasone implants (Ozurdex®) administered
December 2023	Switch from mycophenolate mofetil to methotrexate
February 2024	Last follow-up visit showing persistent chronic-recurrent inflammatory activity

## Discussion

This case illustrates several clinically important aspects of VKH disease. First, the patient presented with rapidly progressive poliosis shortly after an initial episode of anterior uveitis, before a diagnosis of VKH disease had been established. Although integumentary manifestations are classically described during the chronic stage of the disease, they may occasionally constitute an early diagnostic clue and should prompt careful evaluation for VKH disease, particularly in young patients presenting with bilateral ocular inflammation [1,6].

Another notable feature was the absence of neurological or auditory prodromal manifestations. Classical descriptions of VKH disease emphasize a prodromal stage characterized by headaches, meningismus, tinnitus, hearing loss, or vertigo before the onset of overt ocular inflammation [1,6]. However, the revised diagnostic criteria recognize incomplete forms of VKH disease in which only ocular and integumentary manifestations are present [5]. In such situations, multimodal choroidal imaging becomes particularly valuable for establishing the diagnosis and assessing disease activity.

ICGA played a central role throughout the clinical course of our patient. While fluorescein angiography demonstrated improvement and eventual disappearance of active retinal leakage, ICGA repeatedly revealed persistent hypocyanescent lesions consistent with ongoing stromal choroidal inflammation. These findings support previous reports highlighting ICGA as one of the most sensitive techniques for detecting subclinical choroiditis and monitoring therapeutic response in VKH disease [6,7]. The persistence of choroidal inflammatory activity despite apparent retinal quiescence may explain the recurrent relapses observed during follow-up.

The disease course was characterized by progression toward a chronic-recurrent phenotype despite treatment with topical corticosteroids, systemic corticosteroids, mycophenolate mofetil, adalimumab, methotrexate, and repeated intravitreal dexamethasone implants. Several factors may have contributed to this evolution, including delayed initiation of systemic immunosuppression after the initial inflammatory episode, the intrinsically aggressive nature of the disease, and periods of suboptimal adherence to biologic therapy. Although temporary control was achieved with local corticosteroid treatment, recurrent inflammatory activity persisted clinically and angiographically.

This case highlights the importance of recognizing early integumentary manifestations such as poliosis, performing prompt multimodal choroidal assessment, and maintaining sustained suppression of inflammation throughout follow-up. Early diagnosis and rigorous monitoring remain essential to reduce the risk of progression toward chronic-recurrent VKH disease.

## Conclusions

This case illustrates the diagnostic value of early poliosis as a clinical clue to VKH disease, even in the absence of neurological or auditory prodromal manifestations. Despite aggressive multimodal treatment, including systemic corticosteroids, immunomodulatory therapy, biologic therapy, and repeated intravitreal corticosteroid implants, the patient rapidly evolved toward a chronic-recurrent disease course with repeated inflammatory relapses. Early recognition of integumentary manifestations, prompt multimodal choroidal assessment, close monitoring, and sustained treatment adherence remain essential to optimize long-term outcomes in VKH disease.
